# Gene expression profiling within the spleen of *Clostridium perfringens-*challenged Broilers fed antibiotic-medicated and non-medicated diets

**DOI:** 10.1186/1471-2164-10-260

**Published:** 2009-06-07

**Authors:** Aimie J Sarson, Ying Wang, Zhumei Kang, Scot E Dowd, Yang Lu, Hai Yu, Yanming Han, Huaijun Zhou, Joshua Gong

**Affiliations:** 1 Guelph Food Research Centre, Agriculture and Agri-Food Canada, Guelph, Canada; 2 Department of Poultry Science, Texas A &M University, College Station, USA; 3 Research and Testing Laboratories and Medical Biofilm Research Institute, Lubbock, USA; 4 Nutreco Canada Agresearch, Guelph, Canada

## Abstract

**Background:**

*Clostridium perfringens *(Cp) is a Gram-positive anaerobic bacterium that causes necrotic enteritis (NE) in poultry when it overgrows in the small intestine. NE disease has previously been controlled through the use of growth-promoting antibiotics. This practice was recently banned in European countries, leading to significantly increased incidence of NE threatening the poultry industry. Control strategies and technology as substitutes to dietary antibiotics are therefore urgently required. To develop the substitutes, it is important to understand host immune responses to Cp infection. However, the knowledge is still lacking. We therefore investigated gene expression profiles within immunologically-relevant tissue, the spleen, in order to identify factors that are involved in immunity to NE and have potential as therapeutic targets.

**Results:**

Use of a 44 K Agilent chicken genome microarray revealed significant up-regulation of many immune-associated genes in Cp-challenged chickens, including *galectin 3*, *IFNAR1*, *IgY-receptor*, *TCRγ*, *granzyme A*, and *mannose-6-P-R*, which were subsequently validated by quantitative PCR assays. Functional annotation of differentially expressed genes was conducted using the High Throughput Gene Ontology Functional Annotation database. Medicated and Non-medicated chickens had similar annotation profiles with cell activities and regulation being the most dominant biological processes following Cp infection.

**Conclusion:**

Broiler chickens demonstrated an intricate and holistic magnitude of host response to Cp challenge and the development of NE. Although the influence of dietary antibiotics appeared to be less significant than the disease process, both had a considerable impact on the host response. Markers previously identified in intestinal inflammatory diseases of other species, including humans, and indicators of enhanced antibody responses, appeared to be involved in the chicken response to Cp challenge. The significance in host immune responses of immune mediators identified from the present study warrants further studies to verify their functions during NE development and to determine their potential application to control NE disease.

## Background

*Clostridium perfringens *(Cp) is an environmentally dominant anaerobic bacterium, which upon ingestion and overgrowth, can cause intestinal inflammation and necrotic tissue damage, wherein the collective disease pathology is termed necrotic enteritis (NE). The bacterium is Gram-positive, producing spores and a variety of toxins. Cp strains are further classified into subtypes A-E based on the type of toxin they produce (a, b, o and i) and the degree of NE that is resulted, although additional toxins, such as beta2 toxin (*cpb2*), enterotoxin (*cpe*), and necrotic enteritis toxin B-like toxin (*netB*), were recently discovered [1-3]. Sub-clinical NE in poultry is caused by Cp type A and occasionally by type C. Alpha (α)-toxin production has been considered to be to be a major virulence determinant associated with NE disease [4-6]. This was recently challenged by a study of an α-toxin mutant that retained full virulence in a chicken NE model [7]. NetB toxin was also shown to be critical for the production of NE, although not all NE isolates were found to possess the *netB *gene [3]. Although primarily thought to be involved in virulence in humans and piglets, beta (β)-toxin has been suggested to not have great impact on the outcome of NE in chickens, based on the expression of the toxin gene in healthy challenged chickens [8]. Sub-clinical Cp infection in chickens and turkeys also manifests as macroscopic lesions in the small intestine, as well as the caeca, liver, bursa of Fabricius, and kidney [9]. Although sub-clinical Cp infection is commonly observed and only leads to decreased growth performance and mild focal necrosis of the intestinal mucosa of chickens, NE is quite discernably a disease with systemic impact and can lead to acute mortality rates reaching 50% [10]. The severity of NE outbreaks is realized in reports of Cp prevalence in the intestinal tract of poultry ranging from 75% to 95%, with up to 84% of processed poultry meat being Cp positive [11].

Infection with Cp is not the sole factor for the development of NE disease. Predisposing factors such as intestinal damage caused by coccidial pathogens, dietary proteins, and dietary carbohydrate proportions and properties have also been shown to strongly influence the incidence of NE in broilers [12,13]. Since the induction of NE is described as multi-faceted, broad-spectrum growth-promoting antibiotics have primarily been implemented to control the incidence of the inflammatory disease in poultry to date. The recent ban on the use of growth-promoting antibiotics in European food animal production has led to more prevalent Cp infection in poultry and increased outbreaks of NE, threatening the poultry industry [14]. As such, development of strategies to control NE disease has become urgent for both the industry and research communities. Different approaches can be taken for controlling Cp infection, including improving the management of production systems, such as controlled diets and environmental factors, targeting Cp through the control of cell proliferation or toxin production, and enhancing chicken immune defence systems. However, to date, no single "silver bullet" has been invented to control the disease in the field as efficiently and cost-effectively as the prophylactic use of antibiotics. Due to the incompletely characterized profile of Cp-related immune mechanisms, a progressive approach to pursuing prophylactic measures would be to compare the immune response in previously well-characterized antibiotic-medicated chickens, to that of birds that have not been medicated, so that the difference in protective mechanisms may be identified.

Controlling NE disease through probiotic bacteria has been tested previously. Colonization of Cp in the chicken intestine was suppressed when chickens were administered *Bacillus subtilis *prior to Cp challenge [15]. Similarly, a commercial *Lactobacillus*-based probiotic product (All-Lac XCL) was reported to reduce NE-associated mortality from 60% to 30%, but not the degree of NE lesions [16]. Vaccination could be another means to prevent NE in poultry. Previous studies have demonstrated that other animal species including mice, piglets, and calves showed a reduction in the disease prevalence after vaccination with *Clostridium *toxins [17-19]. Protection against sub-clinical NE was also reported in broilers when the chickens were vaccinated with Cp type A and type C toxoids [20]. More recently, virulent and avirulent Cp strains and respective secreted proteins were tested for their ability to orally immunize chickens [6,21]. Virulent Cp isolates protected chickens from subsequent virulent challenge, whereas the avirulent isolate did not [21]. As indicated by serum and intestinal antibody responses, Cp proteins significantly protected broiler chickens against mild Cp infection, whereas only a limited number of proteins offered protection against more severe challenge [6]. Further studies are required to identify true antigens that are able to fully protect chickens from Cp infection.

Gut-associated pathogens have been shown to induce cytokine gene expression changes during systemic infection in chickens, prevalent during various aspects of pathogenesis and immune response, including cellular or tissue entry, immune cell recruitment and pathogen clearance [22]. For example, infection with different *Salmonella *species, has been repeatedly shown to regulate both chemokine and cytokine expression across infection time points [22,23]. Similarly, chicken immune cell cultures infected with various *Eimeria *species have shown a wide array of cytokine responses in cells derived from genetically-defined lines of chickens, demonstrating the influence of host genetic background on susceptibility to disease [24,25]. In many cases, these types of studies are correlated with quantification of pathogen load and descriptions of disease pathogenesis, signs and gross lesions. In the case of Cp infection, our group has previously described bacterial quantification and toxin gene expression in relationship to NE lesion score [26]. However, in comparison to other gut-associated pathogens described above, there is little knowledge about the chicken immune response to Cp infection. The limited reports include recombinant chicken IL-18 used as an adjuvant to stimulate antibody-mediated immune response to chicken pathogens, including Cp α-toxoid [27]. Recently, Collier et al. [28] reported an increase in gene expression of ileal *IL-4*, *IL-10 *and *IFN-γ *in Cp infected chickens when compared with uninfected birds, which coincided with increased intestinal NE lesions and mortality after 2–4 days post-challenge. In addition, Park et al. [29] investigated the immune response of chickens co-infected with *Eimeria maxima *(EM) and Cp by examining the gene expression of a limited number of cytokines and chemokines with quantitative PCR assays. The intestinal expression of a panel of cytokine and chemokine genes following EM/Cp co-infection showed repression of pro-inflammatory interleukin genes (e.g. *IL-12*, *IL-17*) and up-regulation of *IL-8 *and *IL-10 *compared with single infection of Cp or EM. Although expression of many cytokine genes seemed to be strongly induced or repressed in Cp-only treated chickens compared with uninfected birds, no statistical analyses of direct comparisons were presented [29].

The recent development of chicken DNA microarrays, including both cDNA [30] and oligo arrays [31], offered us an opportunity to investigate global gene expression profiling of host response to Cp infection. By using the low-density chicken immune cDNA array [30], we first examined the gene expression profiles in the spleen of broilers experimentally infected with *C*. *perfringens *and found that a more than one immune response pathway was targeted in the host response to Cp infection, including the differential expression of genes within the MHC class I and II and apoptosis pathways [32]. Since oligo arrays are generally of better specificity, sensitivity, and reproducibility than cDNA arrays [33], we revisited the same spleen samples with the 60-mer 44 K chicken whole genome custom array manufactured using the Agilent Technology [31] for a more comprehensive study of the host response to Cp infection. The chickens used for these microarray analyses were from the same infection experiment that investigated the relationships of cell proliferation and α-toxin gene expression of Cp in the chicken intestine with the development of NE lesions [26].

Although Cp infection and NE lesions occurred in the small intestine of chickens, *C*. *perfringens *has been detected in the spleen of infected chickens [28]. Our preliminary data also support this observation (unpublished data). Given this fact and that we were interested in both the local response in the intestine and systemic immune response for defining protective mechanisms against NE, we chose the spleen as our first effort to study the host response to Cp infection. In addition, in mice, intestinal commensal bacteria have been shown to be contained within the gut-associated lymphoid tissues whereas, bacterial pathogens have been shown to break the barrier suggested to be created by the mesenteric lymph nodes (MLN) and are thus found in other lymphoid organs [34]. Considering that Cp can be present in the gastrointestinal (GI)-tract of chickens without causing NE disease, the difference between birds that succumb to disease and those that may harbour the bacteria, could be due to a similar mechanism in chickens whereby once the pathogen reaches systemic lymphoid organs, the host response and disease outcome maybe drastically different than when contained within the GI-tract. Therefore, in the present study, we have analyzed a whole-genome expression profile within the spleen of Cp-infected chickens compared with uninfected birds within the context of antibiotic treatments at a series of time points following the infection.

## Methods

### Bacterium

A Type A strain of *C. perfringens *was grown in Mueller-Hinton broth or on Mueller-Hinton agar containing 5% (vol/vol) sheep blood at 37°C within an anaerobic atmosphere (85% N2, 10% CO2, and 5% H2).

### Experimental animals

The chickens used in the present study were the same birds for previous publications studying gene expression of α-toxin in the chicken intestine [26] and host response to Cp infection with a low-density chicken immune cDNA array [32]. Broiler chickens (Ross × Ross) were reared under the guidelines of the Canadian Council on Animal Care. Six-hundred one-day-old chicks were equally divided among 12 pens (50 birds/pen). Each pen was assigned to one of two dietary treatments: (i) a typical all-vegetable starter diet (Shur-Gain; Nutreco Agresearch Canada) containing zinc bacitracin (55 mg/kg) or (ii) the same diet without bacitracin. The first day of the trial was designated day 0 post-hatch. On day 18, birds were challenged for 16 h with C. *perfringens *(10^7 ^CFU/g feed, 40 g feed/chicken) through the diet after 8 h of starvation. Twelve birds (2 birds per pen) randomly selected from each group were euthanized before and after clostridial challenge daily for five days, which were designated days 0, 1, 2, 3, and 4 post-challenge (D0, D1, D2, D3, and D4 PI), respectively. Spleens were collected from 12 birds of each group for total RNA isolation. RNA samples from D0, D1, D2, and D4 PI birds were used to compare antibiotic-medicated to non-medicated chickens at each time point using microarray analyses.

### RNA extraction

Spleen tissue was homogenized using a Tissue Miser (Fisher Scientific, Houston, TX). Total RNA was isolated from each homogenized tissue using Trizol extraction method as described by the manufacturer (Invitrogen, Carlsbad, CA). DNA was removed from the samples using TURBO DNA *free*™ Kit (Ambion, Austin, TX) according to the manufacturer's protocol. The RNA quantity and purity were determined by NanoDrop ND-1000 spectrophotometer at 260/280 nm (Nano Drop Technologies, Wilmington, Delaware). The integrity of total RNA was assessed with an Agilent Bioanalyzer 2100 and RNA 6000 Nano LabChip Kit (Agilent Technologies, Palo Alto, CA). The RNA Integrity Numbers (RINs) for the samples were obtained. Only RNA samples with RIN values of 6, or higher, were used for further analysis.

### cDNA and cRNA preparation

The cDNA for quantitative PCR analysis was synthesized from 1 μg purified RNA using random hexamers and the Superscript II First Strand cDNA Synthesis kit (Invitrogen, Burlington, Ontario). cRNA for microarray hybridization was prepared as described previously [31]. Briefly, a 500 ng of aliquot of total RNA was reverse transcribed into cDNA using the Low RNA Input Fluorescent Linear Amplification Kit (Agilent Technologies, Palo Alto, CA). Synthesized cDNA was transcribed into cRNA and labelled with either cyanine 3 or cyanine 5-labelled nucleotide (Perkin Elmer, Wellesley, MA). Labelled cRNA was purified with RNeasy Mini columns (Qiagen, Valecia, CA). The quality of each cRNA sample was verified by total yield and specificity calculated based on NanoDrop ND-1000 spectrophotometer measurement (NanoDrop Technologies).

### Microarray experiment design

The design for the present study is similar to our recent report with the low-density chicken immune array [32], except that the 44 K Agilent chicken genome oligo microarray was used. The microarray has been submitted to the National Center for Biotechnology Information Gene Expression Omnibus database under the following accession number: GPL4993. To account for any bias inherent to the fluorescent dyes, a dye swap was performed such that within each treatment group (e.g. Medicated) half of the replicates were labelled with Cy3 and the other half were labelled with Cy5 at each time point. There were six hybridizations performed between Medicated and Non-medicated replicates at D0, D1, D2, and D4 PI, wherein two birds from each pen were pooled within each group.

### Microarray hybridization and analysis

Microarray hybridizations were carried out on labelled cRNAs with specificity greater than 8 using the *in situ *Hybridization Kit Plus (Agilent Technologies). Arrays were incubated at 65°C for 17 h in Agilent's microarray hybridization chambers and subsequently washed according to the Agilent protocol. Arrays were scanned at 5 μm resolution using GenePix Personal 4100A (Molecular Devices Corporation, Sunnyvale, CA). Auto Photomultiplier tube (PMT) gains were adjusted to obtain a ratio of Cy3 and Cy5 channels intensities between 0.95 and 1.05. The signal intensity of all features on each image was quantified by Genepix pro 6.0 software (Molecular Devices Corporation, Downingtown, PA) for further analysis.

The signal intensity of each expressed gene was globally normalized (LOWESS) [35] using the R statistics program and presented on a natural log scale. Microarray data was submitted as GEO series GSE14684 and GSE14759 to Gene Expression Omnibus. A mixed model that included the fixed effects of dye (Cy3 and Cy5), treatment, time, array, and all interactions among treatment and time was used to identify differentially expressed genes between treatments, at the 0.1% significance level using SAS (SAS Institute, Cary, NC). Microarray hybridizations were used to compare Medicated to Non-medicated chickens at each time point (e.g. Medicated vs. Non-medicated on D0 PI) and between time points within treatments (e.g. D0 PI vs. D1 PI in Medicated birds). *P *value and fold changes between each comparison for each gene were calculated. False discovery rate (FDR) (q values) was calculated by R program according to Benjamini and Hochberg's method [36].

Functional annotation of the biological processes involving significantly differentially expressed genes was carried out using an unreleased version of the High Throughput Gene Ontology Functional Annotation Toolkit (HTGOFAT, ). This program was utilized to assign updated Gene Ontology numbers [37], Enzyme Commission numbers [38], and mappings to Kyoto Encylopedia of Genes and Genomes (KEGG) Pathways [39]. Statistics related to over-representation of functional categories were performed using a Fisher Exact statistic methodology [40]. In brief, differentially expressed genes (*p *< 0.001) were selected and separated based on direction of expression (i.e. up- or down-regulated). Data mining to PubMed IDs was performed within HTGOFAT using experimental conditions or terms (e.g. chicken and *Clostridium*) that co-occur with gene names and symbols that are represented in each dataset. Subsequent mapping and clustering was carried out using the Database for Annotation, Visualization and Integrated Discovery (DAVID) [41].

### Quantitative PCR

The quantitative PCR was performed on a Stratagene MX3005 thermal cycler with brilliant SYBR green Q-PCR Master Mix (Stratagene, La Jolla, CA). cDNA was diluted 10-fold, and 1 μl of each diluted sample was added to a 25-μl reaction, also containing 12.5 μL of 2× master mix, 150 nM of each primer, and 30 nM ROX reference dye. Cycling parameters were as follows: 10 min at 95°C, then 40 cycles of 95°C for 30 s, annealing temperature for 30 s, and 72°C for 30 s, and extension for 2 min at 72°C. Gene-specific annealing temperatures are outlined in Table 1. Standard curves were generated by amplifying 3 technical replicates of a serial dilution of plasmid DNA. One plasmid from the serial dilution was used as the PCR calibrator when running biological replicates. The PCR amplification efficiency (E) was calculated using the slope of the standard curve, E = 10^(-1/slope)^. Statistical significance was determined by unpaired T test using mean and SEM of the biological replicates within treatment groups (*p *< 0.05).

**Table 1:** Primer sequence, annealing temperature [AT] (degrees Celsius – °C) and fragment length [FL] (base pairs – bp) for genes amplified using Q- PCR.

Gene Name	Forward Primer	Reverse Primer	AT (°C)	FL (bp)
*Galectin 3*	CTGGATACCCAGGTGCCTAT	AGTACGGTGCAGTTGGTCCT	55	180
*IFNAR1*	CTTTGGGATCCTCGTTCTGA	GCAACACAGGAGGCTTTCAT	55	201
*Mannose-6-P-R*	AAGCTAATGAGAATGAAACGGAGTG	CATCTGGGATGGTTAACACTTCATC	60	180
*MCG24*	TCTGTACAAATTCTGCAGGTCGAAA	AGAGATTCACATTACCTCTTGCCAA	55	222
*TCRγ*	CACATGACACACAAGGCAGCA	TAGCACAGAGAGCAGGAGAGCTTAT	55	225
*Granzyme A*	TGGGTGTTAACAGCTGCTCATTGC	GGGGAATGACTTTCACAGCGCTA	52	213
*IgY R*	AGGAGAACCCGGCCACAC	ATCTGACCACTGCCAGCCA	60	180

## Results

### Microarray analyses

#### Effect of bacitracin on the expression of genes

**Figure 1:**
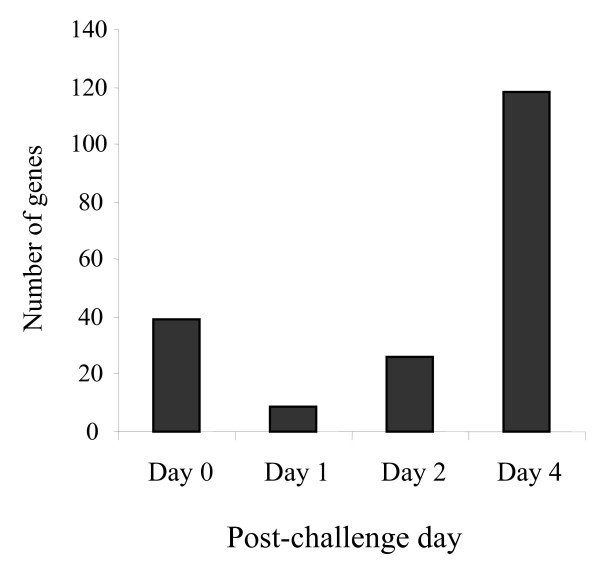
**Numbers of differentially expressed genes between Medicated and Non-medicated groups of birds at each examined time point**. Medicated and Non-medicated groups of chickens represent the birds on diets containing bacitracin (55 ppm) or no antibiotics. Expression data were calculated by mixed model analysis of mean signal intensity minus median background intensity acquired from the Agilent 44 K chicken microarray (*p *< 0.001).

The gene expression data were compared in order to identify the differentially expressed genes between Medicated and Non-medicated groups of chickens at each time point before and after clostridial challenge, including D0, D1, D2, and D4 PI. Few changes were observed at each time point, however the greatest divergence between antibiotic-Medicated and Non-medicated treatment groups occurred on D4 PI for all array results, where D4 PI showed 118 differentially expressed genes with significant changes (*p *< 0.001) (Figure 1). The remaining time points showed less than half the number of genes with significant changes, in which D0 PI (pre-challenged baseline), D1 PI and D2 PI had 9–39 differentially expressed genes between treatment groups (Figure 1). The proportion of genes that were expressed more in Medicated birds than Non-medicated birds was different at each time point. On D0 PI, Medicated group had higher expression of 28% of differentially expressed genes than Non-medicated birds. On D1 PI, all of the genes showed higher expression in the Medicated group compared to the Non-medicated group. On D2 PI, equal numbers of the differentially expressed genes showed higher expression in the Medicated groups and Non-medicated groups. Similarly, on D4 PI 43% of the differentially expressed genes showed higher expression in the Medicated group than the Non-medicated group. Of all the genes differentially expressed with significant changes between Medicated and Non-medicated groups, immune-mediating genes were rarely identified and no genes were commonly expressed at all time points when comparing differentially expressed genes on D0, D1, D2 and D4 PI.

#### Temporal genes expression changes

**Figure 2:**
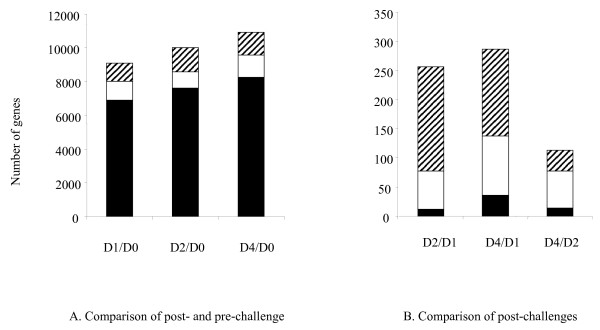
**Numbers of differentially expressed genes in temporal comparison of Medicated and Non-medicated groups of chickens**. **(A) **Comparison of post- and pre-challenges. Number of differentially expressed genes between pre – and post-challenge time points. (**B) **Comparison of post-challenge on different days. Number of genes unique to Medicated (hatched bars), unique to Non-medicated (open bars) treatment groups and number of genes in common for both Medicated and Non-medicated groups (black bars) are represented. Expression data were determined by mixed model analysis of mean signal intensity minus median background intensity measured from Agilent 44 K chicken microarray (*p *< 0.001). Medicated and Non-medicated groups of chickens represent the birds on diets containing bacitracin (55 ppm) or no antibiotics.

There were 8,000 to 11,000 genes differentially expressed in the birds before and after clostridial challenge, regardless of different time points and the treatment of bacitracin (Figure 2A). Within each time point, the majority of genes were significantly expressed in both the Medicated and Non-medicated groups (black bars). Less genes, yet seemingly similar numbers (941–1348) were significantly expressed and unique to either the Medicated (hatched bars) or Non-medicated groups (open bars) (Figure 2A). A comparison of post-challenge time points showed that there was a much greater difference in the number of differentially expressed genes between D1, D2, and D4 PI (Figure 2B). The number of genes in common for Medicated and Non-medicated groups (black bars) was 12–36 genes, representing a much smaller proportion than in the pre- vs. post-challenge comparison. In the comparison of post-challenged birds, genes unique to Medicated (hatched bars) and Non-medicated (open bars) birds dominated the host response with the Medicated group showing a larger significant expression profile during D1 vs. D2 and D1 vs. D4 comparisons than the Non-medicated group. The pattern was reversed when a comparison was made between D2 and D4 PI.

#### Categorical annotation of differentially expressed genes

**Figure 3:**
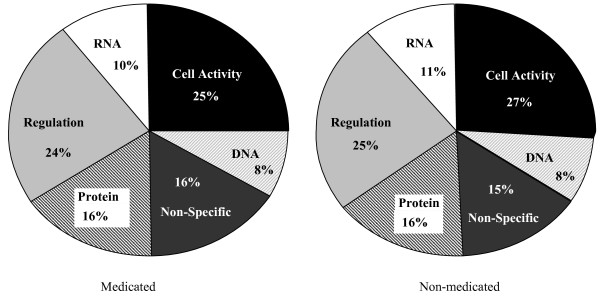
**Comparison of proportions of functional categories within treatment groups resulting from GO Annotation**. Number of functional categories within biological processes defined by Go Annotation using an unreleased version of the High Throughput Gene Ontology Functional Annotation Toolkit (HTGOFAT, ) applied to differentially expressed genes (*p *< 0.001) in Medicated and Non-medicated birds for all time points combined.

Annotation of all significant genes was carried out based on gene sequence association with known biological processes, including those associated with metabolics of chicken RNA, DNA, protein and cell activity and regulation, and some that were not able to be specifically classified. These categories were chosen based on frequency within the dataset after summarizing the full annotation profile, as described in Additional file 1. Medicated and Non-medicated groups had near identical annotation profiles, where cell activity and regulation were the most dominant biological processes during the time course of Cp infection (Figure 3). The differentially expressed genes that were not in common between Medicated and Non-medicated groups over the time course of infection were fairly dispersed in a homogenous manner, with the number of annotated genes ranging from 28.7–32.5% of total number of genes displaying significant expression. The number of immune-specific annotated genes was a much smaller proportion of the total number of significant genes (1.74–2.18%) (Table 2). Of the differentially expressed immune-specific genes, we chose a small number to be investigated for treatment and temporal expression patterns as well as for technical validation by quantitative PCR.

**Table 2:** Numbers of genes with known functions differentially expressed before and after clostridial challenge within Medicated and Non-medicated treatment groups of chickens.

				Number of Genes per Treatment Group	
Post-/pre-challenge			% of Total	Medicated	Non-medicated

D1/D0 PI	Total Number of Genes	2205			
	Number of Annotated as General Function	633	28.7	310	323
	Number of Annotated as Immune Function	48	2.18	26	22

					

D2/D0 PI	Total Number of Genes	2351			
	Number of Annotated as General Function	763	32.5	472	291
	Number of Annotated as Immune Function	41	1.74	29	12

					

D4/D0 PI	Total Number of Genes	2679			
	Number of Annotated as General Function	819	30.6	461	358
	Number of Annotated as Immune Function	48	1.79	26	22

#### Relevant genes regulated during CP infection

**Figure 4:**
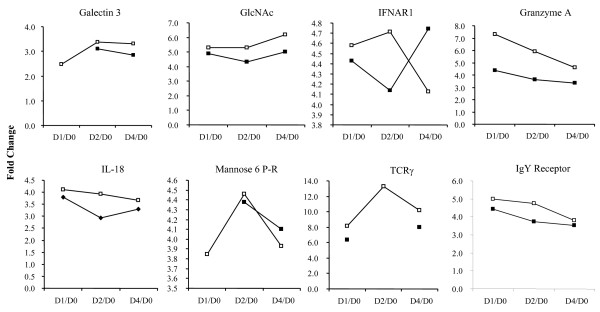
**Temporal expression acquired by comparing post- to pre-challenged chickens in Medicated and Non-medicated groups**. Microarray expression data represented as fold changes by comparing each time point (D1 PI [D1], D2 PI [D2], D4 PI [D4]) in a ratio to D0 PI (D0), indicated for both Medicated (open squares) and Non-medicated (shaded squares) treatment groups. Expression data were determined by mixed model analysis of mean signal intensity minus median background intensity acquired from the Agilent 44 K chicken microarray (*p *< 0.001).

Expression patterns were observed from functionally-relevant chicken genes in both microarray analysis and quantitative PCR validation. Specifically, genes involved in inflammation, antibody response, antigen recognition, apoptosis, and immune-associated metabolic processes showed significant regulation after clostridial challenge. Figure 4 displays the results of microarray analysis of some potential target genes. In general, the expression of all the listed genes was up-regulated in response to clostridial challenge, regardless of the treatment with bacitracin. *IL-18*, *IgY Receptor*, *granzyme A*, *TCR-γ*, *GlcNAc*, and *galectin 3 *had higher expression in Medicated birds than Non-medicated groups at all time points, when normalised to pre-challenged birds. Exceptions of this observation occurred only in the comparison of D4 to D0 PI with *IFNAR1 *and *mannose-6-phosphate receptor (mannose-6-P-R)*. Among different temporal patterns, the up-regulation of *GlcNAc *and *galectin 3 *expression increased during post-challenge days, while *granzyme A*, *IgY Receptor*, and *IL-18 *demonstrated less up-regulation post-challenge. *IFNAR1, mannose-6-P-R, and TCR-γ *showed either increased or decreased up-regulation among different post-challenge days. The up-regulation of most genes after clostridial challenge exceeded 3-fold changes. *TCR-γ *had the highest changes (near 14-fold), while *galectin 3 *exhibited the lowest (more than 2-fold). Although some expression patterns were similar, these could not be attributed to one of the above- mentioned immune function over the others.

### Quantitative PCR verification

Based on the microarray results, genes with a previously defined function and reported correlation to intestinal inflammation or Cp pathology were selected for validation by quantitative PCR. These included the genes encoding Galectin 3, IFNAR1, IgY-R, TCRγ, granzyme A, MCG-24, and Mannose-6-P-R, which were differentially expressed on D1, D2 and D4 PI, when compared with the birds before the challenge (D0 PI). By comparison to *β-actin *(internal control), the expression level of selected genes was determined. In parallel to the microarray analyses, quantitative PCR expression ratios were established by comparing normalised relative expression at each time point after clostridial challenge to that before the challenge (D0 PI) within each treatment group. Up to six comparisons were conducted for each of the genes. Quantitative PCR assays were able to reproduce 60–100% of expression patterns of each gene examined that had been revealed by the microarray analyses (Table 3).

**Table 3:** Validation of microarray results with quantitative PCR assays.

Gene Name	Post-/pre- challenge	Direction of Fold Change in Expression Data		Validation Success
			
		Medicated	Non-medicated	
	D1/D0	+	+	
*Granzyme A*	D2/D0	+	+	6/6 (100%)
	D4/D0	+	+	

	D1/D0	+	+	
*IFNAR1*	D2/D0	+	+	5/6 (83%)
	D4/D0	+	*	

	D1/D0	+	N/A	
*Galectin 3*	D2/D0	+	+	3/5 (60%)
	D4/D0	*	*	

	D1/D0	+	N/A	
*Mannose 6-P-R*	D2/D0	+	+	4/5 (80%)
	D4/D0	+	*	

	D1/D0	+	+	
*TCRγ*	D2/D0	+	N/A	5/5 (100%)
	D4/D0	+	+	

	D1/D0	+	+	
*MCG24*	D2/D0	N/A	N/A	2/3 (66.7%)
	D4/D0	N/A	*	

	D1/D0	+	+	
*IgY R*	D2/D0	+	+	5/6 (83%)
	D4/D0	*	+	

N/A: No significant difference between time points was determined from microarray analysis and no quantitative PCR validation was therefore performed.

*Direction of fold change was conflicting between microarray and quantitative PCR results.

## Discussion

Recently, we reported the use of a low-density cDNA chicken immune-specific microarray to investigate the host immune response to Cp infection by examining gene expression profiles in spleens from experimentally infected chickens [32]. Our results indicated that a more than one immune response pathway was targeted, including the differential expression of genes within the MHC class I and II and apoptosis pathways. Very recently, a 44 K chicken whole genome custom oligo array was developed, which was manufactured with the Agilent Technology [31]. This allowed us to expand our research by compensating for the size limitation of the previous array and thus to more robustly investigate the host response to Cp infection. Importantly, the genes that were previously identified by our group as functionally relevant in the spleen of Cp-challenged chickens (e.g. MHC class I and II family members) were also shown to be differentially expressed using the large-scale array, confirming the technical integrity of both experiments and the biological importance of the gene families.

Upon obtaining the large set of statistically significant gene expression data derived from the analysis of the 44 K microarray, further and extensive data mining and annotation were required to interpret and correlate the results. Numerous reports of expression data annotation have been previously described, in order to assess the large data sets that are considered to be the fruition of high-throughput methodologies. Annotation is necessary because typical methods used to filter the data to a manageable load, such as clustering, can further complicate the dataset, as opposed to converge the output. The Gene Ontology Consortium is a collection of databases for a variety of organisms, which is commonly used to annotate gene expression based on existing knowledge of biological function and a limited functionally-derived vocabulary. This method is organised in a hierarchy of key words including molecular function, biological process, and cellular component [42]. Biological function can then be applied to relevant pathways, through databases such as KEGG (Kyoto Encyclopedia of Genes and Genomes). However, these databases are not nearly as well-developed as the previously mentioned GO databases, suggesting that gaps in the annotation process exist [42]. Furthermore, when studying species that have less known gene functionality (*e.g*. the chicken) compared with the more widely-studied human and mouse, the annotation process can be more challenging. Nonetheless, as shown by the present study (Figure 3 and Additional file 1), GO annotation did provide a multitude of biological processes potentially involved in the chicken response to Cp infection and NE development, and identified fewer processes that are solely affected by feeding the birds a Medicated diet (bacitracin) compared to the Non-medicated. One might note that this process did not narrow the results to a manageable size. On the contrary, the GO annotation established a data categorization that leads to a new realm of questions regarding the roles of these processes in Cp infection. Moreover, the results of GO annotation required a user-imposed, arbitrary clustering of the processes into more general terms, as demonstrated in Figure 3.

Specific target genes were selected from the list of genes that were differentially expressed to a significant degree for further studies to infer biological function. Selection was based on GO annotation and previous reports of disease pathology associated with NE in chickens or other species, and other diseases that manifest similarly. Specifically, NE lesions have been observed in the small intestine, caeca, liver and kidney of both chickens and turkeys [9], and the small intestine is the main site and characterized by thin friable walls indicative of exacerbated inflammation [43-45]. Histopathological studies in NE diseased birds have revealed sloughed intestinal epithelium, heterophil infiltration into the lamina propria and enterocyte necrosis as indicated by the presence of matrix metalloproteinase (MMP)-2 enzyme [46,47]. In humans, perivascular infiltration with polymorphonuclear, mononuclear, and eosinophil cells, in the presence of intestinal necrosis, is observed in cases of necrotizing enteritis [48]. The occurrence of eosinophilia, manifesting as a local type I hypersensitivity response, suggests that this mechanism may also be present in the chicken during the course of NE lesion development. Interestingly, our results from the present study concur with both controlled and excessive inflammatory processes, as well as an antibody receptor gene up-regulation potentially related to an eosinophil response. Regardless of the comparison made between Medicated and Non-medicated chickens, with the aim of deciphering protective and compromised immune responses during the course of NE development, the largest difference in expression profiles were observed before and after clostridial infection irrespective of antibiotic treatments. Thus during the process of correlating biological significance with gene expression data, the focus was shifted primarily to CP-infected birds in comparison to non-infected birds.

Of the genes identified as biologically-relevant from functional annotation, *galectin 3*, *IFNAR1*, *IgY R *and TCR-γ appear to be significantly relevant to the mechanism of development of NE. Expression profiles of *IgY R *and *TCR-γ *can be speculated to be correlated with innate immunity responses and eosinophil-regulated inflammation, respectively, whereas *galectin 3 *and *IFNAR1 *biological functions throughout CP infection are primarily based on previous characterization of inflammatory disease processes. Type I interferons (IFNs) are typically described as important mediators of the anti-viral cytokine response and more generally speaking, mediators for a pro-inflammatory response [49]. For example, type I IFNs are produced upon bacterial activation of macrophages, which initiates an up-regulatory autocrine-type production of IFNs. Specifically, IFN-α and IFN-β, whose expression patterns are mediated by availability of receptors IFNAR1 and IFNAR2, induce IFN-γ and IL-18 production [50]. In concert, our results indicate up-regulated *IFNAR1 *expression in Medicated birds on (D1 and D2 PI) compared to D0 PI baseline and the same time point ratios in the Non-medicated groups. At the latest time point, the expression ratio is reversed, in which Non-medicated chickens had the highest level of expression on D4 PI compared to pre-challenged chickens and compared to the same ratio in the Medicated group of chickens. This increase in inflammation at early time points in Medicated birds may suggest a correlation between the protective antibacterial effect of IFNAR1 on D1 PI and D2 PI. However, the rise in *IFNAR1 *expression on D4 PI in Non-medicated birds may suggest a shift from a protective immune response to an exacerbated, lesion-forming inflammation as observed in Cp-infected chickens developing NE. Importantly, IL-18 expression was also up-regulated in clostridial challenged chickens on D1-D4 PI compared with pre-challenged controls for both the Medicated and Non-medicated groups, with the Medicated group showing the highest level of expression of all.

Galectin 3 is a β-galactoside-specific lectin that is expressed on intestinal epithelial cells (IELs) [51]. Aside from facilitating adhesion, galectin 3 is strongly involved in inflammatory processes and mRNA expression has been observed in IELs from patients with Crohn's disease and bowel information, colon carcinoma and colitis [51]. Suggesting a connection to the antibody-mediated response, serum from Crohn's disease patients was shown to contain anti-galectin-3 IgG antibodies. Moreover, galectin 3 acts as receptor for IgE, facilitating the up-regulation of IgE production in atopic patients [52]. Specifically, B cells expressing surface IgE expressed higher levels of galectin 3 than B cells of other phenotypes [53]. The relationship between IgE, galectin 3 and eosinophil activity in allergy-induced inflammation is such that eosinophils, which are activated upon IgE production, are recruited to sites of inflammation by galectin 3 [54]. Similarly, other members of the galectin family have been shown to stimulate production of pro-inflammatory cytokines under intestinal inflammatory conditions [55,56] suggesting an integral role of galectins in inflammatory host responses, with emphasis on the intestinal induction site.

In general, the antibody response in chickens is similar to mammals [57,58]. Avian IgY shares properties with both mammalian IgG and IgE. Although in most cases, IgY molecule is still referred to as chicken IgG, as it appears to be functional equivalent to mammalian IgG. Despite lacking evidence that birds produce the mammalian equivalent of IgE, there is similarity in NE pathogenesis in chickens compared to other IgE-mediated host responses to gut-associated pathogens inducing intestinal inflammation in mammals [59-61], suggesting that our observation on *IgY R *gene induction and previously reported IgY antibody production following exposure to Cp proteins may be representative of IgE activity. To validate the hypothesis, further functional studies with chicken eosinophils, antibody titres and hypersensitivity are required.

Lastly, TCR-γ expression was shown to be consistently up-regulated in infected chickens, regardless of antibiotic treatment, indicative of the involvement of γδ-T cells in the host response to Cp infection. Specifically, γδ-T cells, which are dominant at mucosal surfaces, have been described to interact with innate immunity cells including mammalian dendritic cells [62] and chicken NK-like cells [63] during cellular activation, and presumably at the outset of pathogenic infection. In chickens, NK-like cell activity has been shown in intestinal epithelial lymphocyte populations potentially containing chicken γδ-T cells [64], and γδ-T cells have been reported within the caecum [65]. Although further studies are required to determine the role of this T cell sub-population in chickens during Cp infection, our results suggest γδ-T cells may represent a previously unreported innate immunity mechanism to combat NE disease.

## Conclusion

In conclusion, the data acquired from the present study demonstrate the intricate and holistic magnitude of chicken host response to Cp challenge and the development of NE. Although the influence of dietary antibiotics appeared to be less significant than the disease process, both had a considerable impact on the host response. Identification of target genes relevant to Cp infection and antibiotic effect warrants further functional studies that may lead to the determination of molecular mechanisms underlying the chicken response and to the development of effective control strategies for NE disease in poultry.

## Abbreviations

α: alpha; β: beta; CFU: colony forming units; Cp: Clostridium perfringens; cpb2: beta 2 toxin; cpe: enterotoxin; D: day; DAVID: Database for Annotation, Visualiation and Integrated Discovery; EM: Eimeria maxima; FDR: False Discovery Rate; GO: gene ontology; h: hour; HTGOFAT: High Throughput Gene Ontology Functional Annotation Toolkit; IEL: intraepithelial lymphocyes; Ig: immunoglobulin; IL: interleukin; IFN: interferon; K: 1,000; KEGG: Kyoto Encyclopedia of Genes and Genomes; MHC: major histocompatibility complex; NE: necrotic enteritis; netB: necrotic enteritis toxin B-like toxin; PCR: polymerase chain reaction; PI: post-infection; ppm: parts per million; RIN: RNA integrity number; SEM: standard error of mean.

## Authors' contributions

JG, HZ, and AJS designed research. YW, ZK, YL, HY, YH, and AJS performed research. HY and YH coordinated the chicken trial. AJS, YW, HZ, and JG analyzed and interpreted data. SED contributed a new analytic tool; and AJS and JG prepared the manuscript.

## Authors' informations

Yang Lu was a visiting graduate student to J. Gong's laboratory.

## Supplementary Material

Additional File 1 **Number of differentially expressed genes assigned to biological function categories based on GO Annotation**. Number of genes up-regulated, shown as positive numbers on X-axis, and down-regulated, shown as negative numbers on X-axis, within each time point comparison, were classified within biological processes defined by GO Annotation using an unreleased, chickens-specific version of the High Throughput Gene Ontology Functional Annotation Toolkit (HTGOFAT, ). Time points compared within microarray hybridizations are shown as D1 vs. D0 PI (black bars), D2 vs. D0 PI (open bars), and D4 vs. D0 PI (hatched bars) where Non-medicated birds are indicated by NM and Medicated birds are indicated by M. (A) Number of differentially expressed genes with a role in cell activity in Non-medicated and Medicated birds, respectively; (B) Number of differentially expressed genes with a role in cellular regulation in Non-medicated and Medicated birds, respectively; (C) Number of differentially expressed genes associated with DNA processes in Non-medicated and Medicated birds, respectively; (D) Number of differentially expressed genes associated with RNA processes in Non-medicated and Medicated birds, respectively; (E) Number of differentially expressed genes associated with protein processes in Non-medicated and Medicated birds, respectively; (F) Number of differentially expressed genes possessing biological function that was not classified within the above-noted categories in Non-medicated and Medicated birds, respectively. Click here for file
