# Supplementary material for: Gene expression profiling within the spleen of Clostridium perfringens-challenged Broilers fed antibiotic-medicated and non-medicated diets
Source: BMC Genomics. 2009 Jun 7;10:260. doi: 10.1186/1471-2164-10-260 (PMC2703656; doi:10.1186/1471-2164-10-260)
Supplement: Additional File 1 — Number of differentially expressed genes assigned to biological function categories based on GO Annotation. Number of genes up-regulated, shown as positive numbers on X-axis, and down-regulated, shown as negative numbers on X-axis, within each time point comparison, were classified within biological processes defined by GO Annotation using an unreleased, chickens-specific version of the High Throughput Gene Ontology Functional Annotation Toolkit (HTGOFAT, ). Time points compared within microarray hybridizations are shown as D1 vs. D0 PI (black bars), D2 vs. D0 PI (open bars), and D4 vs. D0 PI (hatched bars) where Non-medicated birds are indicated by NM and Medicated birds are indicated by M. (A) Number of differentially expressed genes with a role in cell activity in Non-medicated and Medicated birds, respectively; (B) Number of differentially expressed genes with a role in cellular regulation in Non-medicated and Medicated birds, respectively; (C) Number of differentially expressed genes associated with DNA processes in Non-medicated and Medicated birds, respectively; (D) Number of differentially expressed genes associated with RNA processes in Non-medicated and Medicated birds, respectively; (E) Number of differentially expressed genes associated with protein processes in Non-medicated and Medicated birds, respectively; (F) Number of differentially expressed genes possessing biological function that was not classified within the above-noted categories in Non-medicated and Medicated birds, respectively. [file 1471-2164-10-260-S1.ppt]

## Slide 1
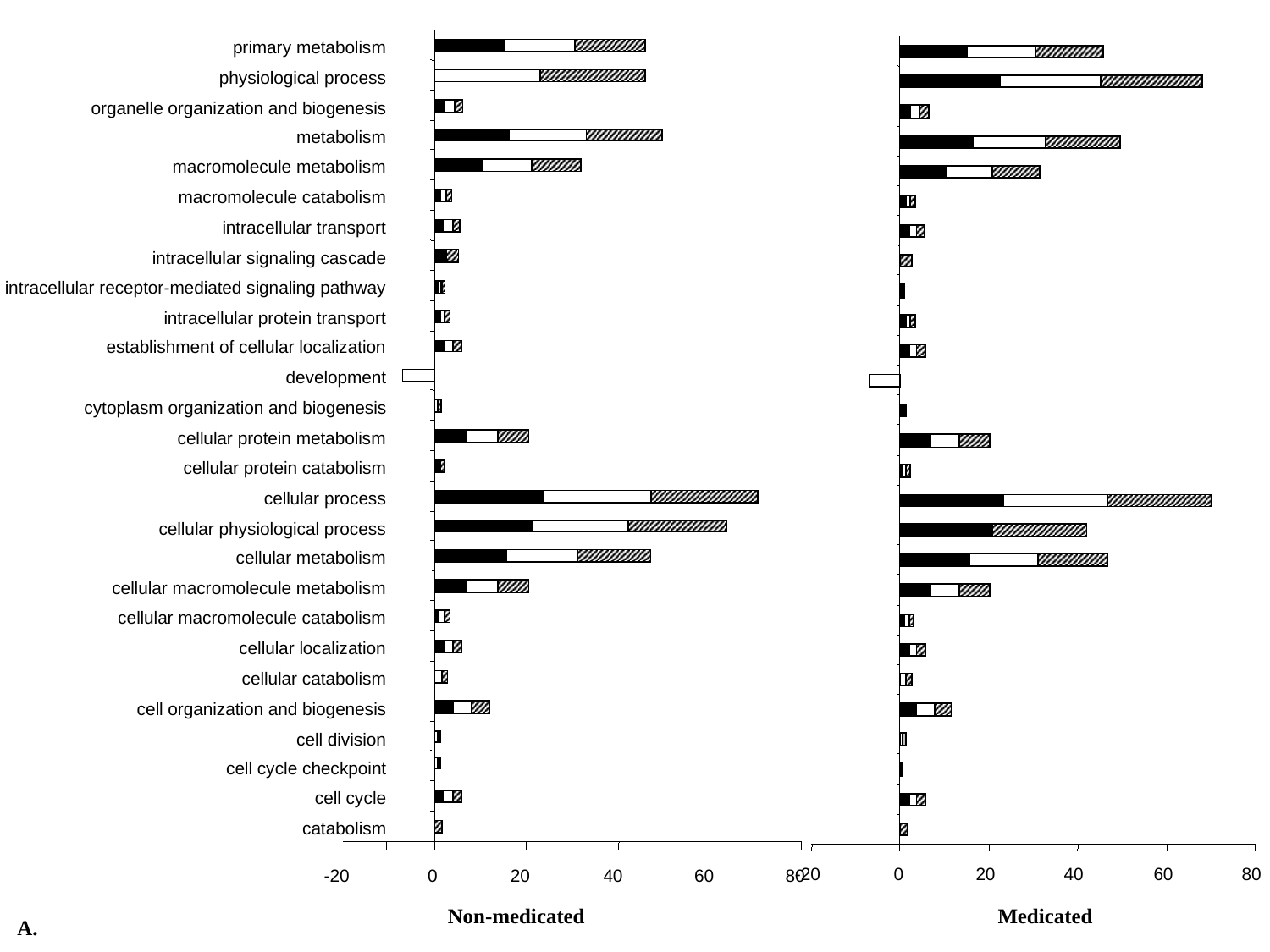

-20
0
20
40
60
80
primary metabolism
physiological process
organelle organization and biogenesis
metabolism
macromolecule metabolism
macromolecule catabolism
intracellular transport
intracellular signaling cascade
intracellular receptor-mediated signaling pathway
intracellular protein transport
establishment of cellular localization
development
cytoplasm organization and biogenesis
cellular protein metabolism
cellular protein catabolism
cellular process
cellular physiological process
cellular metabolism
cellular macromolecule metabolism
cellular macromolecule catabolism
cellular localization
cellular catabolism
cell organization and biogenesis
cell division
cell cycle checkpoint
cell cycle
catabolism
-20
0
20
40
60
80
Non-medicated
Medicated
A.

## Slide 2
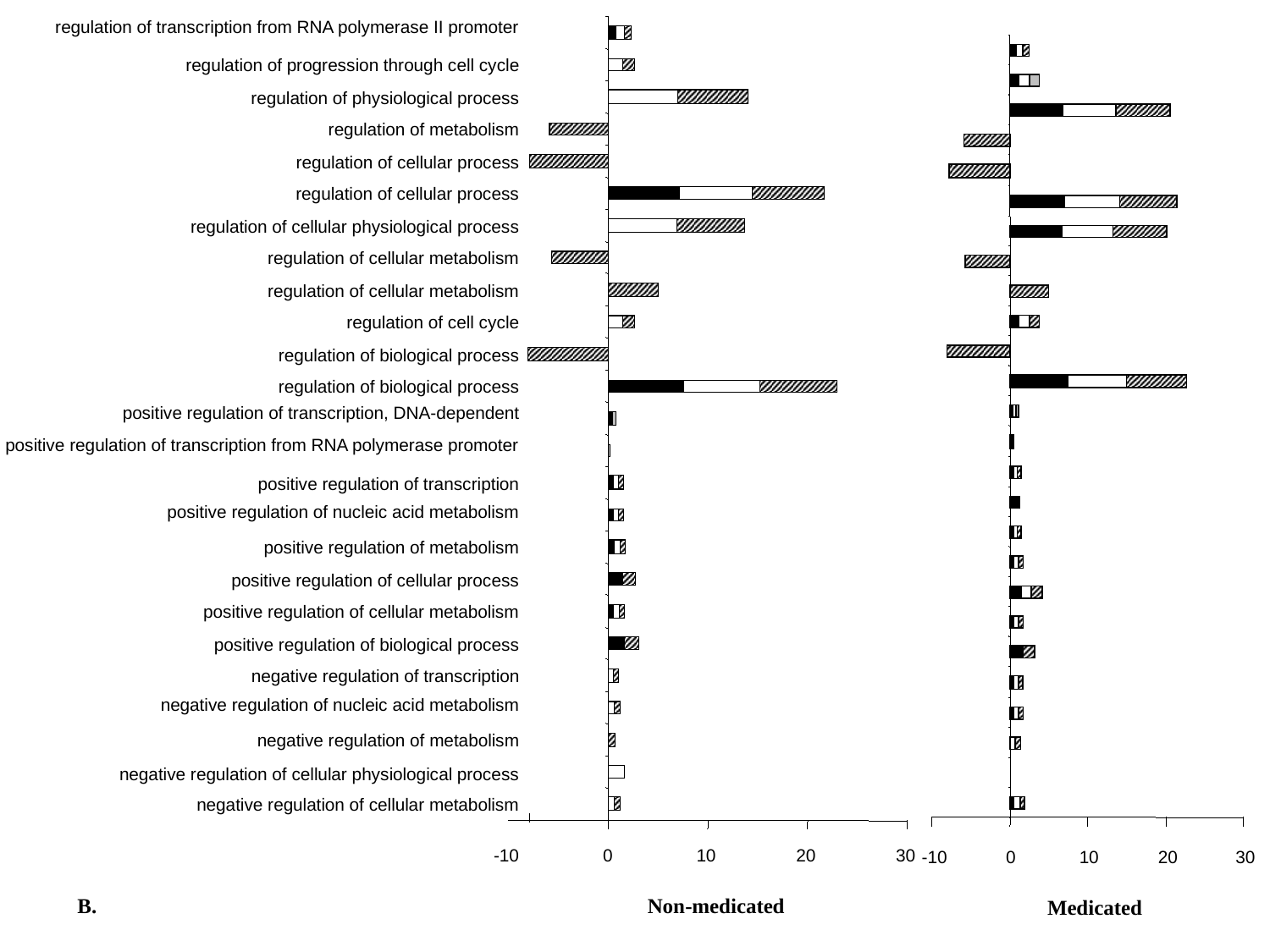

regulation of transcription from RNA polymerase II promoter
regulation of progression through cell cycle
regulation of physiological process
regulation of metabolism
regulation of cellular process
regulation of cellular process
regulation of cellular physiological process
regulation of cellular metabolism
regulation of cellular metabolism
regulation of cell cycle
regulation of biological process
regulation of biological process
positive regulation of transcription, DNA-dependent
positive regulation of transcription from RNA polymerase promoter
positive regulation of transcription
positive regulation of nucleic acid metabolism
positive regulation of metabolism
positive regulation of cellular process
positive regulation of cellular metabolism
positive regulation of biological process
negative regulation of transcription
negative regulation of nucleic acid metabolism
negative regulation of metabolism
negative regulation of cellular physiological process
negative regulation of cellular metabolism
-10
0
10
20
30
-10
0
10
20
30
B.
Non-medicated
Medicated

## Slide 3
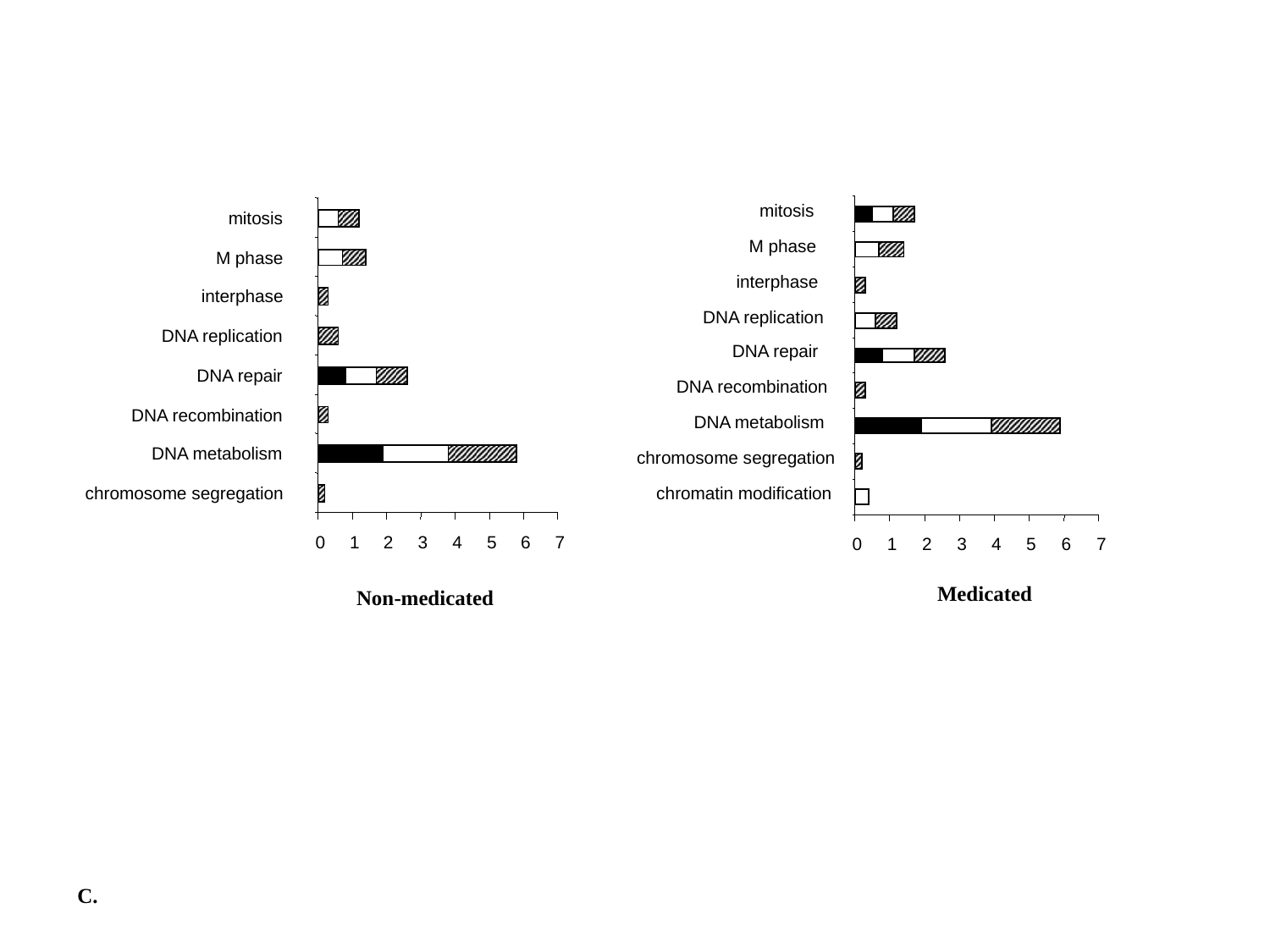

0
1
2
3
4
5
6
7
0
1
2
3
4
5
6
7
mitosis
M phase
interphase
DNA replication
DNA repair
DNA recombination
DNA metabolism
chromosome segregation
chromatin modification
mitosis
M phase
interphase
DNA replication
DNA repair
DNA recombination
DNA metabolism
chromosome segregation
Medicated
Non-medicated
C.

## Slide 4
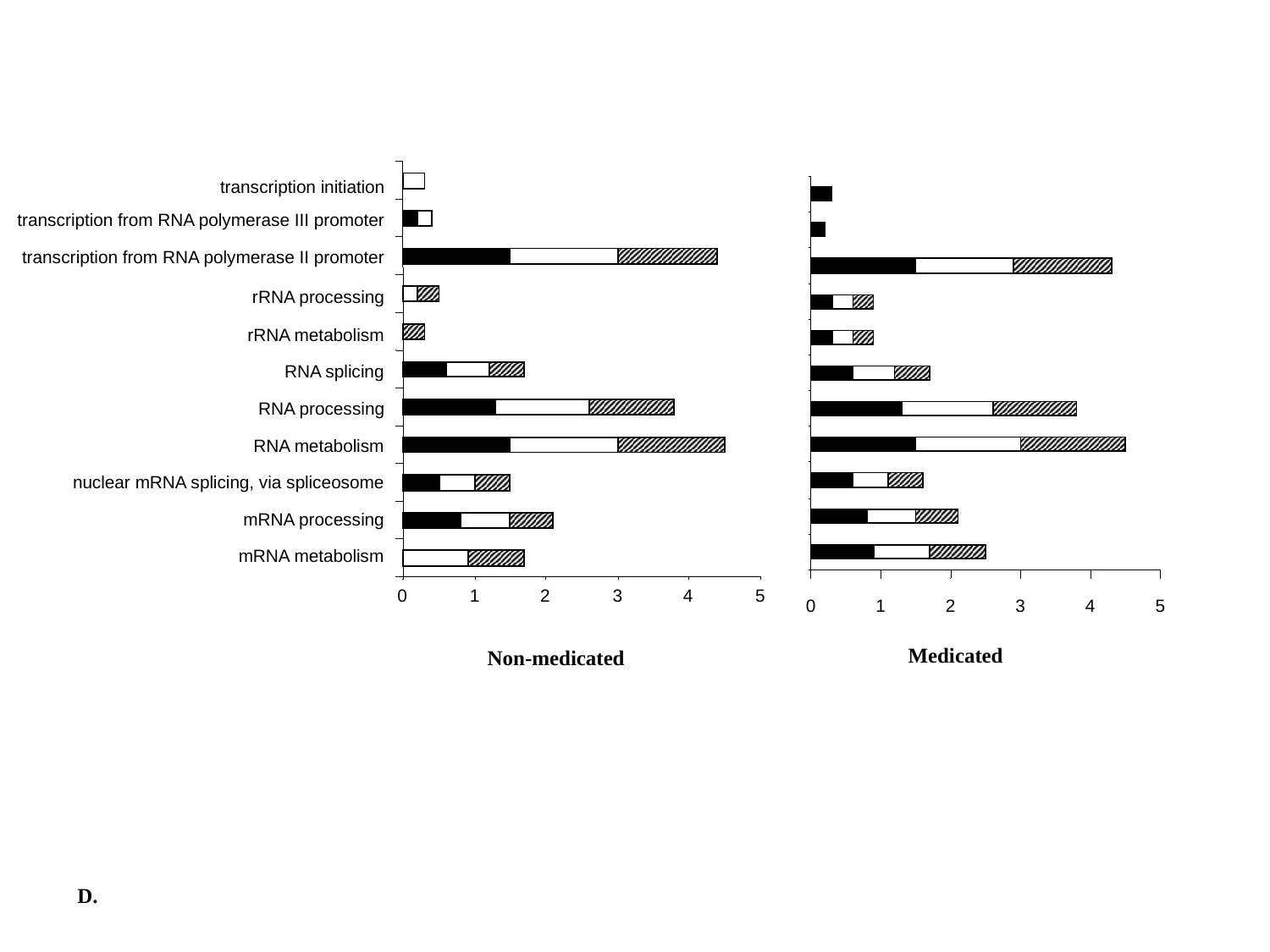

0
1
2
3
4
5
transcription initiation
transcription from RNA polymerase III promoter
transcription from RNA polymerase II promoter
rRNA processing
rRNA metabolism
RNA splicing
RNA processing
RNA metabolism
nuclear mRNA splicing, via spliceosome
mRNA processing
mRNA metabolism
0
1
2
3
4
5
Medicated
Non-medicated
D.

## Slide 5
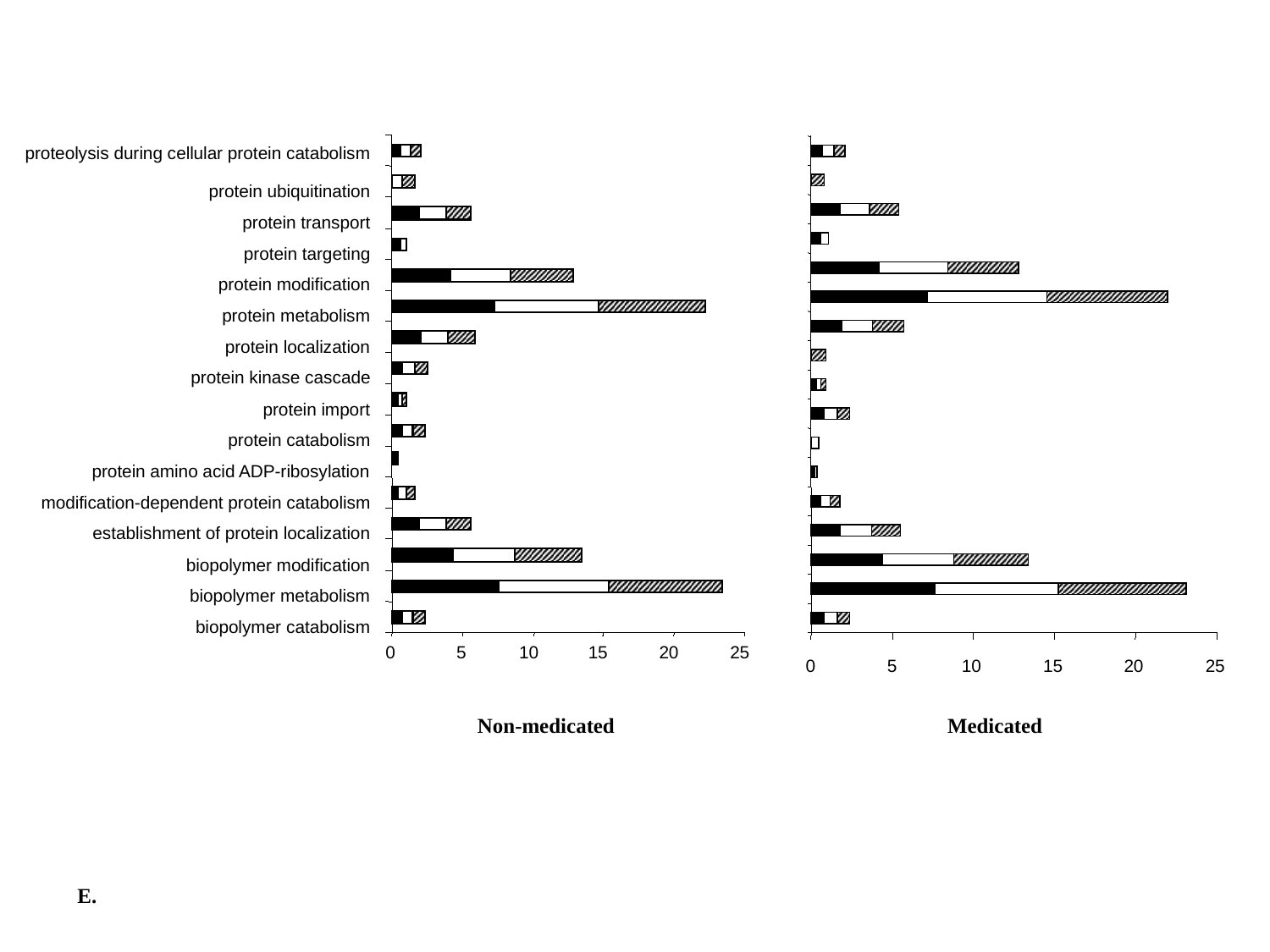

0
5
10
15
20
25
0
5
10
15
20
25
proteolysis during cellular protein catabolism
protein ubiquitination
protein transport
protein targeting
protein modification
protein metabolism
protein localization
protein kinase cascade
protein import
protein catabolism
protein amino acid ADP-ribosylation
modification-dependent protein catabolism
establishment of protein localization
biopolymer modification
biopolymer metabolism
biopolymer catabolism
Non-medicated
Medicated
E.

## Slide 6
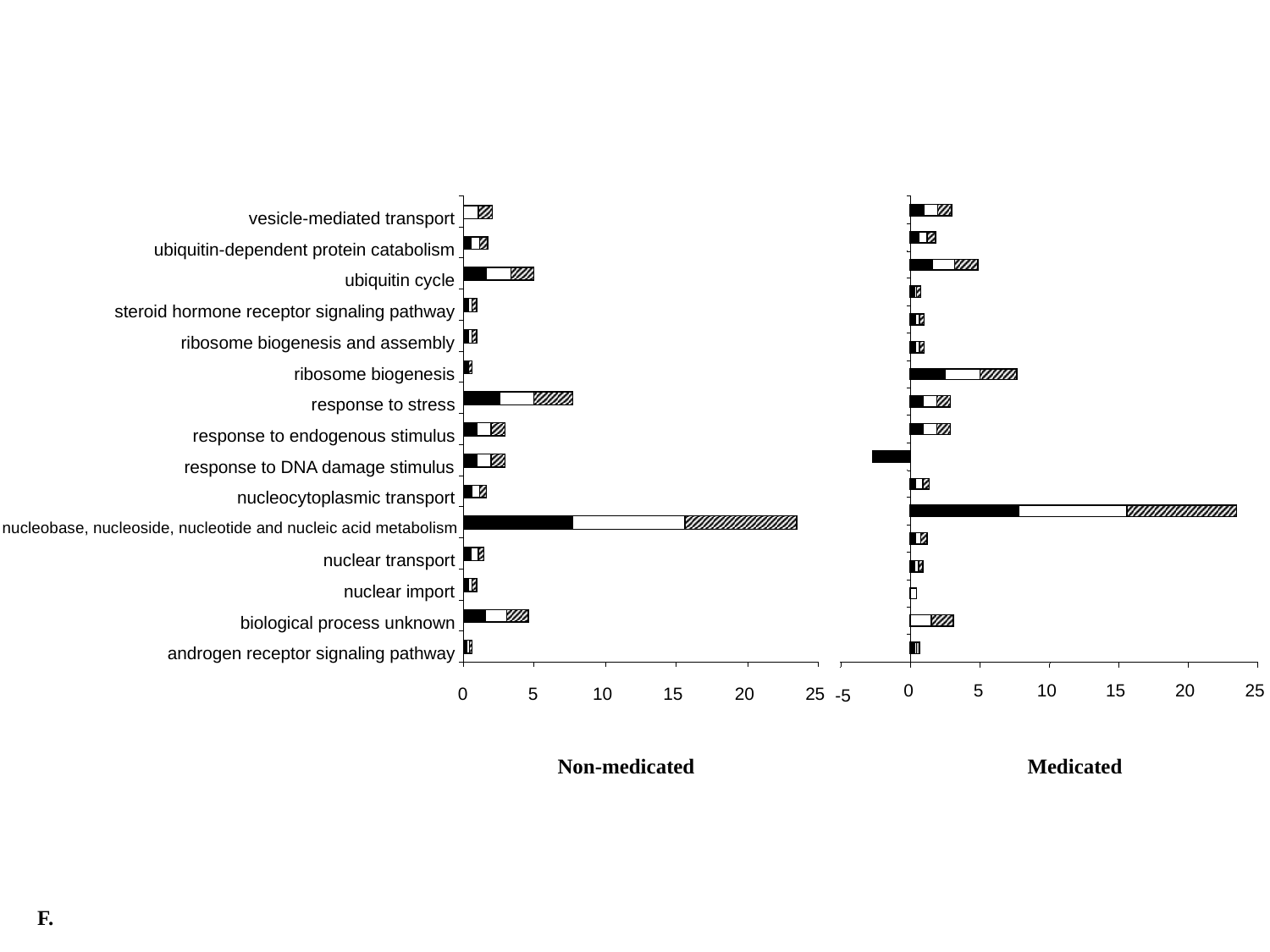

0
5
10
15
20
25
0
5
10
15
20
25
-5
vesicle-mediated transport
ubiquitin-dependent protein catabolism
ubiquitin cycle
steroid hormone receptor signaling pathway
ribosome biogenesis and assembly
ribosome biogenesis
response to stress
response to endogenous stimulus
response to DNA damage stimulus
nucleocytoplasmic transport
nucleobase, nucleoside, nucleotide and nucleic acid metabolism
nuclear transport
nuclear import
biological process unknown
androgen receptor signaling pathway
Non-medicated
Medicated
F.
